# Zika-related adverse outcomes in a cohort of pregnant women with rash in Pernambuco, Brazil

**DOI:** 10.1371/journal.pntd.0009216

**Published:** 2021-03-08

**Authors:** Ricardo Arraes de Alencar Ximenes, Demócrito de Barros Miranda-Filho, Ulisses Ramos Montarroyos, Celina Maria Turchi Martelli, Thalia Velho Barreto de Araújo, Elizabeth Brickley, Maria de Fátima Pessoa Militão de Albuquerque, Wayner Vieira Souza, Liana O. Ventura, Camila V. Ventura, Adriana L. Gois, Mariana C. Leal, Danielle Maria da Silva Oliveira, Sophie Helena Eickmann, Maria Durce C. G. Carvalho, Paula F. S. da Silva, Maria Angela Wanderley Rocha, Regina Coeli Ferreira Ramos, Sinval Pinto Brandão-Filho, Marli Tenorio Cordeiro, Luciana Caroline Albuquerque Bezerra, George Dimech, Sandra Valongueiro, Pedro Pires, Priscila Mayrelle da Silva Castanha, Rafael Dhalia, Ernesto Torres Azevedo Marques-Júnior, Laura C. Rodrigues, on behalf of the Microcephaly Epidemic Research Group (MERG)

**Affiliations:** 1 Departamento de Medicina Tropical, Universidade Federal de Pernambuco, Recife, Pernambuco, Brazil; 2 Faculdade de Ciências Médicas, Universidade de Pernambuco, Recife, Pernambuco, Brazil; 3 Instituto de Ciências Biológicas, Universidade de Pernambuco, Recife, Pernambuco, Brazil; 4 Departamento de Saúde Coletiva, Instituto Aggeu Magalhães, Recife, Pernambuco, Brazil; 5 Departamento de Medicina Social, Universidade Federal de Pernambuco, Recife, Pernambuco, Brazil; 6 Department of Infectious Disease Epidemiology, London School of Hygiene & Tropical Medicine, London, United Kingdom; 7 Departamento de Oftalmologia, Fundação Altino Ventura, Recife, Pernambuco, Brasil; 8 Departamento de Cirurgia, Universidade Federal de Pernambuco, Recife, Pernambuco Brazil; 9 UTI Pediátrica, Hospital Universitário Oswaldo Cruz, Recife, Pernambuco, Brazil; 10 Departamento Materno-Infantil, Universidade Federal de Pernambuco, Recife, Pernambuco, Brazil; 11 Departamento de Neurologia, Hospital Universitário Oswaldo Cruz, Recife, Pernambuco, Brazil; 12 Departamento de Infectologia Pediátrica, Hospital Universitário Oswaldo Cruz, Recife, Pernambuco, Brazil; 13 Departamento de Imunologia, Instituto Aggeu Maglhães, Recife, Pernambuco, Brazil; 14 Laboratório de Virologia e Terapia Experimental, Instituto Aggeu Maglhães, Recife, Pernambuco, Brazil; 15 Secretaria Executiva da Vigilância em Saúde, Secretaria de Saúde de Pernambuco, Recife, Pernambuco, Brazil; 16 Departamento Materno-Infantil, Universidade de Pernambuco, Recife, Pernambuco, Brazil; 17 Programa de Pós-graduação em Ciências da Saúde, Universidade de Pernambuco, Recife, Pernambuco, Brazil; Beijing Children’s Hospital, Capital Medical University, CHINA

## Abstract

**Background:**

While Zika virus (ZIKV) is now widely recognized as a teratogen, the frequency and full spectrum of adverse outcomes of congenital ZIKV infection remains incompletely understood.

**Methods:**

Participants in the MERG cohort of pregnant women with rash, recruited from the surveillance system from December/2015-June/2017. Exposure definition was based on a combination of longitudinal data from molecular, serologic (IgM and IgG3) and plaque reduction neutralization tests for ZIKV. Children were evaluated by a team of clinical specialists and by transfontanelle ultrasound and were classified as having microcephaly and/or other signs/symptoms consistent with congenital Zika syndrome (CZS). Risks of adverse outcomes were quantified according to the relative evidence of a ZIKV infection in pregnancy.

**Findings:**

376 women had confirmed and suspected exposure to ZIKV. Among evaluable children born to these mothers, 20% presented with an adverse outcome compatible with exposure to ZIKV during pregnancy. The absolute risk of microcephaly was 2.9% (11/376), of calcifications and/or ventriculomegaly was 7.2% (13/180), of additional neurologic alterations was 5.3% (13/245), of ophthalmologic abnormalities was 7% (15/214), and of dysphagia was 1.8% (4/226). Less than 1% of the children experienced abnormalities across all of the domains simultaneously. Interpretation: Although approximately one-fifth of children with confirmed and suspected exposure to ZIKV in pregnancy presented with at least one abnormality compatible with CZS, the manifestations presented more frequently in isolation than in combination. Due to the rare nature of some outcomes and the possibility of later manifestations, large scale individual participant data meta-analysis and the long-term evaluation of children are imperative to identify the full spectrum of this syndrome and to plan actions to reduce damages.

## Author summary

The ability to meaningfully quantify the absolute and relative risks of Congenital Zika Syndrome is contingent on the accurate identification of ZIKV infections in pregnant women and the long-term follow-up of children at risk. This study builds on the evidence base on ZIKV in pregnancy by not only examining pregnancy and birth outcomes, but also considering later onset manifestations of Congenital Zika Syndrome, conducting a deep and standardized investigation of infant outcomes. In addition, it used unprecedented repeated testing and the use of multiple diagnostic platforms, including qRT-PCR, IgM and IgG3 ELISAs, and PRNTs to identify ZIKV cases. The absolute risk of microcephaly was 2.9%, of calcifications and/or ventriculomegaly was 7.2%, of additional neurologic alterations was 5.3%, of ophthalmologic abnormalities was 7% and of dysphagia was 1.8%. The manifestations presented more frequently in isolation than in combination (i.e., less than 1% of the children experienced abnormalities across all of the domains simultaneously). Although approximately one-fifth of children with confirmed and suspected exposure to ZIKV in pregnancy presented with at least one abnormality compatible with CZS, the manifestations presented more frequently in isolation than in combination

## Introduction

Since the start of the microcephaly epidemic in the Americas in 2015, evidence detailing the consequences of prenatal exposure to Zika virus (ZIKV) has been accumulating. Case reports provided vital descriptions mainly of affected children with severe manifestations [1], and shed light on both common features (e.g., calcifications [2]) as well as organ system-specific signs and symptoms (e.g., cryptorchidism [3]). A constellation of structural birth defects and functional disabilities is now clinically recognizable as Congenital Zika Syndrome (CZS) [4,5], though there are no standard criteria for diagnosing CZS. Key questions persist related to the relative frequency of CZS and its overlapping clinical features.

To date, several cohort studies of ZIKV-infected pregnant women have begun to estimate the risks of adverse pregnancy and birth outcomes after infection [6–13] and consistently estimate the absolute risk of microcephaly to be approximately 5%. However, there is wide variation in the estimated frequencies of other adverse outcomes, ranging from 6 [7] to 46% [6]. These other outcomes mainly include grossly abnormal clinical or brain imaging findings or both [6], often accompanied by neurologic and ocular defects [7]. As methodological issues limit the comparability of the existing findings, the spectrum of abnormalities arising from prenatal exposure to ZIKV and the risk of each of them—either separately or in combination—remains incompletely understood.

Pernambuco in the northeast of Brazil was the epicenter of the microcephaly epidemic that, along with a cluster of other neurological disorders, triggered the World Health Organization’s declaration of a Public Health Emergency of International Concern[14]. At the request of the Brazilian Ministry of Health and the State Health Secretariat, the Microcephaly Epidemic Research Group (MERG) was assembled, bringing together multidisciplinary investigators from several institutions across Pernambuco state and abroad. The MERG was tasked with providing an initial description of microcephalic cases [1], verifying the association between prenatal ZIKV exposure and microcephaly using a case-control study [15,16], and estimating the risks of adverse outcomes using a cohort study of pregnant women.

The pregnancy cohort study, MERG cohort, was designed at the height of the epidemic in coordination with the public health response efforts of the Pernambuco State Health Secretariat. This study adopted a unique approach evaluating the children by different groups of specialists using standardized protocol and forms, on the same day, and quantifying the risks according to the relative evidence for a ZIKV infection in pregnancy, contributing to understanding of the risks and spectrum of CZS. This comprehensive evaluation allowed us to investigate a range of adverse outcomes compatible with the exposure to ZIKV infection during pregnancy.

## Methods

### Ethics statement

The project was approved by the Research Ethics Committee of the Aggeu Magalhães / Fiocruz Research Center, with CAAE: 53240816.4.0000.5190 and followed the ethical procedures recommended for this type of study by Resolution MS/CNS 466/2012. Free and informed written consent was requested for all mothers/legal guardians of children.

### Study design and data collection

Following the identification of the microcephaly epidemic, in December 2015, the Pernambuco State Health Department introduced a surveillance system for pregnant women presenting with rash (Center for Strategic Information on Health Surveillance in Pernambuco; CIEVS/PE). Participants in the MERG cohort were recruited from Pernambuco state’s compulsory notification system for pregnant women with rash. The surveillance protocols of the Pernambuco State Health Secretariat and MERG protocols were harmonized, and some activities shared. Pregnant women with rash were notified to the surveillance program by the health units, and blood samples collected within five days of rash onset (time point 1). At least 14 days after notification, the MERG fieldworkers recruited these women into this study, collected a second blood sample and administered a standardized questionnaire (time point 2). In cases of livebirths, a third blood sample was collected from participating women after delivery (time point 3). Testing at each timepoint was independent of the others (i.e., women could be tested at one point even if they had not been tested in the previous one).

Recruitment commenced in December/2015 and included women residing up to 120km from the Metropolitan Region of Recife. The first blood samples were sent to the Central Laboratory of Public Health in Recife, where were separated and stored at -80°C until further diagnostic testing was performed at the Laboratorio de Virologia e Terapia Experimental of the Fundação Oswaldo Cruz (LAVITE-Fiocruz). The second and third blood samples were separated, stored, and tested at LAVITE-Fiocruz.

At baseline, pregnant women were evaluated in terms of sociodemographic factors (age, race/ethnicity, years of education, social class, and monthly per capita family income), reproductive history (previous pregnancy, children with malformations from previous pregnancies), current pregnancy characteristics (smoking, illicit drug use, delivery mode). For social class we used the new Brazilian Economic Classification Criteria [17], which is based on the pattern of consumption of the Brazilian families. It ranges from A (higher) to E (lower). Smoking and illicit drug use were defined according to the information given by the pregnant women in response to the questionnaire, independently of the gestational age and of the frequency and quantity.

Gestational age at the time of rash onset and delivery was estimated, in order of priority, by first trimester ultrasound, the date of last menstrual period, and any other trimester ultrasound.

### Diagnostic assays

The main exposure, prenatal ZIKV infection, was based on clinical (rash in any form), and laboratory criteria, based on the combination of longitudinal data from nucleic acid amplification tests, serologic assays and plaque reduction neutralization tests, as previously described [18]. Further laboratory tests were performed in the pregnant women with rash to test for TORCH agents and other arboviruses.

#### Molecular assay

Maternal sera were tested for the detection of ZIKV RNA by one-step qRT-PCR using primers and probes previously described [19].

#### Serologic assays

Samples were screened for the detection of ZIKV-specific IgM antibodies by capture-IgM enzyme-linked immunosorbent assay (ELISA) [20]. Maternal sera were also tested for the detection of ZIKV-specific IgG3 anti-non-structural protein 1 (NS1) antibodies using a novel in-house ELISA following a protocol previously described [21]. ZIKV-specific neutralizing activity was assessed in all available maternal sera by PRNT, following a standardized protocol [22] carried out in Vero cells using a virus strain isolated in the study setting: ZIKV (strain Brazil/PE243/2015) [23]. The cut-off value for PRNT positivity was defined based on a 50% reduction in plaque counts (PRNT50). ZIKV-specific neutralizing antibody titers were estimated by four-parameter nonlinear regression using the sigmoidal dose response (variable slope) equation on GraphPad Prism 7.0a. Maternal sera were considered ZIKV-non-negative with PRNT50 titers ≥20, equivocal with PRNT50 titers ≥20 and <100, and ZIKV-positive with PRNT50 titers ≥100. Seroconversion was considered to occur with four-fold rises in PRNT50 titers or with a switch from negative (i.e., <20) to non-negative status (i.e., ≥20). Maternal sera of a subsample was tested for DENV using Panbio Dengue IgG Indirect ELISA.

### Degrees of evidence of ZIKV infection

We categorized maternal ZIKV exposure according to the degree of evidence of infection (See Ximenes et al. 2019 [18] for detailed description). Pregnant women were classified as Positive for ZIKV infection, Suspected or Negative. The positive group was divided into three subcategories (robust, moderate and limited evidence) according to the level of diagnostic evidence. Robust evidence was defined by a positive qRT-PCR, seroconversion, or at least two positive serologic tests in pregnancy or by one positive IgM or IgG3 in pregnancy paired with a non-negative PRNT50 within six months post-pregnancy. Moderate evidence was defined by one positive IgM or IgG3 in pregnancy, an indication of seroconversion by PRNT50 during pregnancy (i.e., either a PRNT50 titer ≥1000 in pregnancy paired with a rise within 2 months post-pregnancy or 4-fold rise in PRNT50 titer from pregnancy to within 2 months post-pregnancy), or an equivocal PRNT50 test result in pregnancy paired with a positive PRNT50 within three months post-pregnancy. Limited evidence of ZIKV infection was defined by a positive PRNT50 in pregnancy or within 6 months post-pregnancy or an indication of PRNT50 seroconversion during the 2 to 3 months post-pregnancy. The Suspected ZIKV infection group included two subcategories (limited evidence of flavivirus and inconclusive results). Limited evidence of a flavivirus before or during pregnancy was defined by a PRNT50 titer between 20 and 100 or a non-negative result (i.e., unspecified titer ≥20) in pregnancy or within 1 month post-pregnancy, providing evidence of infection by an unspecified flavivirus. The inconclusive subgroup included pregnant women who tested IgM negative for ZIKV, but were tested more than three months after the rash started, and women who showed inconsistency in the results of the same test. We considered there to be evidence against ZIKV infection if all tests performed in pregnancy were negative.

### Pediatric assessment

All children born to the mothers of the MERG Pregnant Women Cohort were referred to just one health unit where they were evaluated by different specialists of the MERG team, using standardized protocols especially created for these assessments. Transfontanelle ultrasound (TF-US) was performed within the first six months of life. Priority was given to those children born to mothers with lab evidence of ZIKV infection, independently of the level of evidence, but the evaluation was carried out for all children independently of suspicious of an abnormality. The timing of evaluation was constrained by the operational capacity of the research team, as the Northeast of Brazil was the place where the cases of microcephaly were first identified and Pernambuco was in the epicentre of the epidemic, and the study was designed and conducted when the epidemic was already occurring. This evaluation of the outcomes of the pregnant women cohort was also the baseline evaluation of the MERG Paediatric Cohort.

Pediatricians evaluated infants for malformations or other abnormalities. Microcephaly was defined as a head circumference falling two or more standard deviations below the mean for age and sex. For infants born with gestational age ≥37 weeks, head circumference was evaluated using the WHO Anthro software and classified according to the World Health Organization (2007) growth charts [24]. For premature infants, age was corrected for gestational age at delivery and classified using the Intergrowth (2015) growth charts [25]. Cases were classified as having disproportionate microcephaly based on the ratio of head circumference to length [2]. Where head circumference in cm was less than ½ length in cm –7 (1/2L-7), microcephaly cases were considered to be disproportionate.

Pediatric neurologists examined children for: seizures, altered consciousness/behavior, localized motor deficits, altered tonus or trophism, and pyramidal signs. Neuroimaging results from the TF-US were assessed for: calcifications, cerebellar hypoplasia or atrophy, agenesis or dysgenesis of the corpus callosum and diffuse cortical atrophy.

Otolaryngologists performed clinical and auditory evaluations, including the short latency auditory brainstem response to stimuli. If the first screening test was abnormal, the test was repeated later. If the second test also indicated hearing loss, the child was referred for further diagnosis.

Speech therapists evaluated infants’ ability to swallow and the stomatognathic system, following a clinical protocol based on the Pediatric Dysphagia Assessment Protocol [26].

Pediatric ophthalmologists and retina specialists from the Altino Ventura Foundation, evaluated children for: visual acuity testing, oculomotility testing, cycloplegic refraction, indirect ophthalmoscopy, and wide-angle fundus imaging (Retcam Shuttle, Clarity Medical Systems, Pleasanton, California, United States of America).

Children were classified as having signs and symptoms consistent with CZS if they had one or more of the following: microcephaly, neuroimaging, neurologic, auditory and ophthalmologic abnormalities.

### Statistical analysis

We estimated the absolute risk and respective 95% confidence interval of microcephaly and other adverse outcomes potentially related to prenatal exposure to Zika virus infection separately for each outcome and for the combined presentation. The analysis was stratified according to the level of laboratory evidence, allowing a sensitivity analyses. We also estimated the relative risk, 95% confidence interval and p-value (Chi-squared test) for the association between trimester of gestation in which the infection occurred, years of education and social class with microcephaly and with any of the other abnormalities. For comparing the clinical features of ZIKV infection we used the Chi-squared test or Fisher’s exact tests for categorical variables and the Mann-Whitney U tests for continuous variables. We used the Stata software (version 14.1 (College Station, Texas, USA)) for the statistical analyses.

## Results

**Fig 1:**
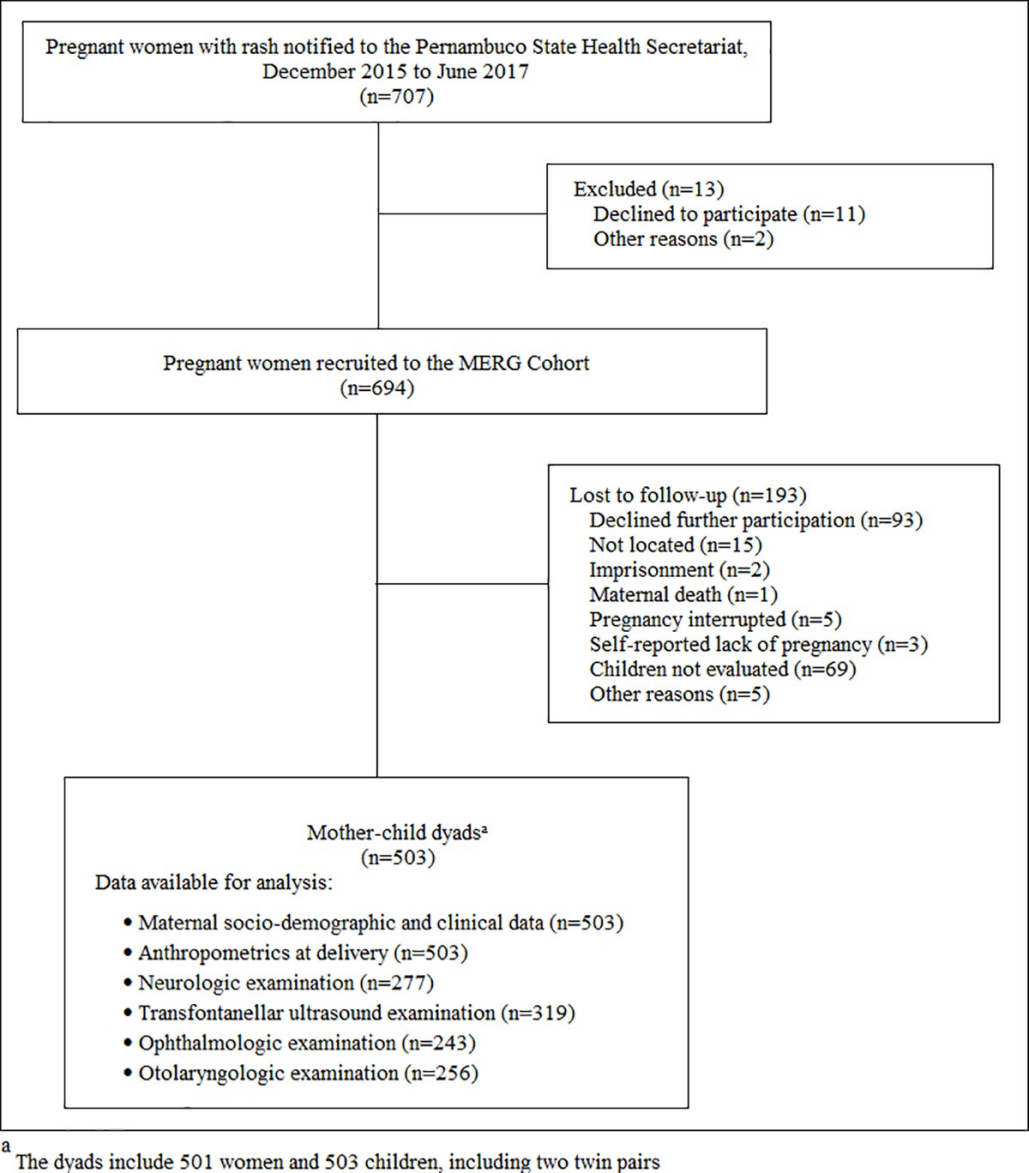
Flow diagram of the MERG Pregnancy Cohort in Pernambuco, Brazil (2015–2017).

A total of 694 pregnant women with rash was recruited between December/2015 and June/2017. Out of the total, 364 (52.4%) women were tested for ZIKV infection after delivery (at time point 3), 240 (34.6%) at 14 or more days after notification (at time point 2) and 90 (13.0%) within five days of rash onset (at time point 1). A total of 503 children born to these women were evaluated sometime after birth (Fig 1). In relation to the exposure status of their mothers, 277 were classified as having some level of laboratory evidence of ZIKV infection during pregnancy, 78 of unspecified Flavivirus and for 19 the results were inconclusive. For 127 there was no laboratory evidence of ZIKV infection (Table 1).

**Table 1:** Characteristics of women with rash in the MERG Pregnancy Cohort in Pernambuco, Brazil (2015–2017).

Variables	Total^a^	N (%)
**Sociodemographic factors**		
** Age, years, Median (IQR)**	501	26.2 (21–31)
** Race/ethnicity**	500	
** **“Preto” (ie, black)		50 (10.0)
** **“Pardo” (ie, mixed race)		335 (67.0)
** **“Branco” (ie, white)		107 (21.4)
** **Other		8 (1.6)
** Years of education**	501	
** **0–8		181 (36.1)
** **11-Sep		263 (52.5)
** **12+		57 (11.4)
** Social Class**^**b**^	501	
** **A, B1, and B2		47 (9.4)
** **C1		90 (18.0)
** **C2		186 (37.1)
** **D and E		178 (35.5)
** Monthly per capita family income, BRL, Median (IQR)**	421	1156 (880–1880)
**Reproductive history**		
** Previous pregnancy**	501	
** **Yes		320 (63.9)
** **No		181 (36.1)
** Children with malformations from previous pregnancies**	501	
** **Yes		12 (2.4)
** **No		278 (55.5)
** **N/A^c^		211 (42.1)
**Current pregnancy characteristics**		
** Smoking**	501	
** **Yes		38 (7.6)
** **No		463 (92.4)
** Illicit drug use**	501	
** **Yes		7 (1.4)
** **No		494 (98.6)
** Delivery mode**	494	
** **Cesarean		227 (46.0)
** **Vaginal		266 (53.8)
** **Forceps		1 (0.2)
** ZIKV diagnosis in pregnancy**	501	
** **ZIKV-positive		277 (55.3)
** ***Robust evidence*, *PCR*		*108*
** ***Robust evidence*, *serology*		*53*
** ***Moderate evidence*, *serology*		*53*
** ***Limited evidence*, *serology*		*53*
** **Unspecified flavivirus-positive		78 (15.6)
** **Inconclusive		19 (3.8)
** **ZIKV-negative		127 (25.3)

^**a**^Numbers varied because of missing values.

^b^Based on the pattern of consumption of the Brazilian families, A being the higher and E being the lower.

^**c**^Not applicable due to abortion, stillbirth, or no previous pregnancy.

The time elapsed between birth and first evaluation had a median of 4.65 months (IQR: 2.9–13.4). There were two foetal deaths, one with a gestational age of approximately 5 months and the other with a gestational age of 34 weeks. Five deaths occurred after delivery, two within one day, one within two months and two after a year. The two latter deaths occurred in children with Down Syndrome. For three of these five children, including those with Down Syndrome, there was a report of severe cardiac malformation.

The sociodemographic characteristics, reproductive history and substance use of the pregnant women are presented in Table 1 and S1 Table.

Rash was referred in the interview by 98% of the women. Approximately 80% informed having had fever, joint pain and fatigue/feebleness. Joint pain, joint swelling and presence of secretion in the eyes when wake up were more frequent among women who had laboratory evidence of ZIKV infection (Table 2).

**Table 2:** Clinical presentation of women with rash at the time of notification by ZIKV diagnostic status in the MERG Pregnancy Cohort in Pernambuco, Brazil (2015–2017)^a^.

Clinical Features	ZIKV-positive	Suspected ZIKV exposure		ZIKV-negative (n = 127)	*P value*^*b*^ *Positive v*. *negative*	*P value*^*c*^ *Positive+Suspected v*. *negative*
	Robust + Moderate + Limited (n = 277)	Unspecified flavivirus-positive (n = 78)	Inconclusive (n = 19)			
	N (%)					
**Rash**	273 (98.6)	78 (100)	17 (84.2)	123 (96.8)	*0*.*27*	*0*.*48*
**Location where rash first appeared**						
** **Head/neck	19 (6.9)	6 (7.7)	0 (0)	4 (3.1)	*0*.*17*	*0*.*19*
** **Torso/back	46 (16.6)	12 (15.4)	1 (5.3)	27 (21.3)	*0*.*26*	*0*.*16*
** **Abdomen	127 (45.8)	38 (48.7)	9 (47.4)	53 (41.7)	*0*.*44*	*0*.*35*
** **Arms	60 (21.7)	18 (23.1)	9 (47.4)	29 (22.8)	*0*.*79*	*0*.*92*
** **Legs	43 (15.5)	10 (12.8)	1 (5.3)	16 (12.6)	*0*.*44*	*0*.*60*
** **Full body	18 (6.5)	7 (9.0)	0 (0)	7 (5.5)	*0*.*70*	*0*.*64*
Duration of rash, days (Median (IQR))	4.5 (3–7)	4 (3–7)	5 (2–15)	5 (3–7)	*0*.*29*	*0*.*23*
** **Fever	220 (80.6)	64 (82.0)	11 (68.7)	97 (78.9)	*0*.*69*	*0*.*71*
** **Myalgia	197 (71.1)	47 (60.3)	10 (52.6)	82 (64.6)	*0*.*19*	*0*.*49*
** **Arthralgia	236 (85.2)	65 (83.3)	12 (63.2)	96 (75.6)	*0*.*02*	*0*.*04*
** **Joint swelling	196 (70.8)	51 (65.4)	9 (47.4)	76 (59.8)	*0*.*03*	*0*.*08*
Retro-bulbar pain	78 (28.2)	18 (23.1)	6 (31.6)	39 (30.7)	*0*.*60*	*0*.*46*
Secretions in the eyes when waking up	20 (7.2)	5 (6.4)	0 (0)	3 (2.4)	*0*.*06*	*0*.*07*
Conjunctivitis	75 (27.1)	16 (20.5)	4 (21.0)	38 (29.9)	*0*.*55*	*0*.*32*
Photophobia	71 (25.6)	15 (29.2)	5 (26.3)	43 (33.9)	*0*.*09*	*0*.*04*
Headache	215 (77.6)	62 (79.5)	14 (73.7)	91 (71.6)	*0*.*19*	*0*.*16*
Vomiting/nausea	117 (42.2)	34 (43.6)	7 (36.8)	52 (40.9)	*0*.*81*	*0*.*80*
Rhinorrhea	37 (13.4)	10 (12.8)	2 (10.5)	10 (7.9)	*0*.*11*	*0*.*11*
Pruritis	232 (83.8)	63 (80.8)	13 (68.4)	108 (85.0)	*0*.*74*	*0*.*49*
Back pain	181 (65.3)	48 (61.5)	8 (42.1)	78 (61.4)	*0*.*44*	*0*.*69*
Fatigue	219 (79.1)	62 (79.5)	13 (68.4)	100 (78.7)	*0*.*94*	*0*.*97*
Pharyngitis	34 (12.3)	10 (12.8)	2 (10.5)	10 (7.9)	*0*.*19*	*0*.*17*
Cough	29 (10.5)	7 (9.0)	2 (10.5)	10 (7.9)	*0*.*41*	*0*.*45*
Abdominal pain	77 (27.8)	17 (21.8)	6 (31.6)	31 (24.4)	*0*.*47*	*0*.*61*

^a^The numbers varied because of the missing values.

^b^Comparison of the ZIKV-positive group versus the ZIKV-negative group: *P* values for categorical variables are from Chi-squared tests or, for variables with cells including ≤5 observations, Fisher’s exact tests; *P* values for continuous variables are from Mann-Whitney U tests.

^c^Comparison of the positive and suspected exposure group (i.e., ZIKV-positive, unspecified flavivirus positive, and inconclusive groups) versus the ZIKV-negative group: *P* values for categorical variables are from Chi-squared tests or, for variables with cells including ≤5 observations, Fisher’s exact tests; *P* values for continuous variables are from Mann-Whitney U tests.

Table 3 shows the absolute risk of the main adverse outcomes according to the level of ZIKV exposure during pregnancy. The columns in bold summarise the information for all positive categories (i.e., robust, moderate, and limited evidence) and the combined positive plus suspected categories (i.e., robust, moderate, and limited evidence plus flavivirus and inconclusive). In addition to absolute risks at first evaluation, we also added prospective information of the risk of presenting with neurologic and ophthalmologic abnormalities and dysphagia at any time during the follow up. Of the 503 pregnant women with rash, 12 cases of microcephaly were identified, one occurring in a child born to a woman with no laboratory evidence of Zika virus infection. Among the 376 women who were ZIKV positive or had suspected ZIKV exposure, the absolute risk of microcephaly was approximately 3% (Table 3). Among these cases, three were disproportionate. Nine had a TF-US performed and no abnormality was observed in 7; in one child cranial haemorrhage grade I was observed and in another, born to the woman with no laboratory evidence of ZIKV infection, alteration of the optic nerve and retina, a benign lump of the subaracnoid space and asymmetry were described. For two children (out of three) with disproportionate microcephaly and TF-US performed, no abnormality was found.

**Table 3:** Absolute risk and 95% confidence interval of Zika related adverse outcomes in the MERG Pregnancy Cohort in Pernambuco, Brazil (2015–2017).

		Positive				Suspected ZIKV exposure			Negative	p-value (all positive x negative)	p-value (all suspected x negative)
	Cases/sample^c^	Robust	Moderate	Limited	Robust + Moderate + Limited	Flavivírus	Inconclusive	Positive + Flavivírus + inconclusive			
**Microcephaly**	12/503	2/162 (1.2) 0.03–4.9	1/28 (3.6) 0.4–23.7	4/88 (4.5) 1.7–11.7	**7/278 (2.5)** **1.2–5.2**	3/78 (3.8) 1.2–11.5	1/20 (5.0) 0.6–32.3	**11/376 (2.9)** **1.6–5.2**	1/127 (0.8) 0.1–5.2	0.453	0.301
**Altered transfontanelle US**^**a**^	18/319	5/84 (5.9) 2.4–13.7	4/24 (16.7) 5.9–38.9	2/56 (4.3) 0.8–13.7	**11/164 (6.7)** **3·7–11·8**	2/46 (4.3) 1.0–16.5	0/18 (0)	**13/180 (7.2)** **3.3–9.6**	5/91 (5.5) 2.3–12.7	0.702	0.590
**Neurologic Abnormalities**^b^											
**At first evaluation**	14/277	6/144 (4.2) 1.9–9.0	0/20 (0)	4/52 (7.7) 2.8–19.3	**10/216 (4.6)** **2.5–8.4**	2/20 (10.0) 2.2–35.5	1/9 (11.1) 0.9–62.6	**13/245 (5.3)** **3.1–8.9**	1/32 (3.1) 0.4–20.9	1.000	1.000
**At any time**	-	-	-	-	**28/216 (13.0)** **9.1–18.2**	-	-	**34/245 (13.9)** **10.0–18.8**	**4/32 (12.5)** **4.5–30.2**	1.000	1.000
**Ophthalmologic Abnormalities**											
**At first evaluation**	20/243	6/143 (4.2) 1.9–9.1	1/20 (5.0) 0.6–32.3	5/24 (20.8) 8.3–43.2	**12/187 (6.4)** **3.7–11.0**	2/18 (11.1) 2.8–38.9	1/9 (11.1) 0.09–62.6	**15/214 (7.0)** **4.2–11.3**	5/29 (17.2) 0.07–36.7	0,118	0,145
**At any time**	-	-	-	-	**14/187 (7.5)** **4.5–12.3**	-	-	**18/214 (8.4)** **5.3–13.0**	**6/29 (20.7)** **9.1–40.4**	0.069	0.097
Abnormal auditory brainstem response											
**At first evaluation**	11^d^/253	7/133 (5.3) 2.5–10.7	1/18 (5.6) 0.6–35.5	2/48 (4.2) 1.0–15.9	**10/199 (5.0)** **2.7–9.1**	1/17 (5.9) 0.7–37.2	0/7 (0)	**11/223 (4.9)** **2.7–8.7**	0/30 (0)	0.367	0.371
**At any time**	-	-	-	-	**10/199 (5.0)** **2.7–9.1**	-	-	**11/223 (4.9)** **2.7–8.7**	**0/30 (0)**	0.367	0.371
**Dysphagia**											
**At first evaluation**	4/256	2/132 (1.5) 0.4–5.9	0/18 (0)	2/50 (4.0) 1.0–15.3	**4/200 (2.0)** **0.7–5.2**	0/17 (0)	0/9 (0)	**4/226 (1.8)** **0.7–4.7**	0/30 (0)	1.000	1.000
**At any time**	-	-	-	-	**6/200 (3.0)** **1.3–6.6**	-	-	**6/226 (2.6)** **1.2–5.8**	**0/30 (0)**	**0.856**	**0.939**

^a^Calcification or ventriculomegaly.

^b^At least one of the following: inappropriate visual response, alteration of tonus or trophism, altered level of consciousness/behavior and convulsions.

^c^The denominator varies according to the missing values for each outcome.

^D^Nine children repeated the test and it was normal on the second evaluation.

Of 263/503 children who had TF-US performed, 30 had some imaging alteration, of which 21 were among non-negative. The absolute risk of any imaging alteration in children born to non-negative mothers was 9.2% and of calcifications or ventriculomegaly 5.7%, but these risks were similar among the offspring of negative mothers. Of the five children who had imaging alteration and were born to ZIKV negative mothers, two were born to Chikungunya positive mothers, one being PCR positive and, the other being IgM positive; two were born to mothers negative for TORCH, Dengue and Chikungunya and one to a mother who was not tested for TORCH.

Microcephaly and TF-US suggestive of CZS occurred independently of the trimester in which there was a probable Zika infection (Table 4).

**Table 4:** Gestational age of rash and risk of Zika related adverse outcomes in the Zika positive group of the MERG Pregnancy Cohort in Pernambuco, Brazil (2015–2017).

Trimester of rash	Number of children^b^	Number of cases (%)	95%-CI	Relative risk (95%-CI)	p-value
**Microcephaly**	278	7			
** **First	38	1 (1.8)	0.2–10.0	Reference	-
** **Second	86	3 (2.2)	0.7–6.6	1.33 (0.14–36.1)	1.000
** **Third	154	7 (3.8)	1.8–7.8	1.73 (0.22–13.6)	1.000
**Any of the abnormalities**^**a**^	185	45			
** **First	32	8 (25.0)	12.5–43.7	Reference	-
** **Second	63	7 (11.1)	5.3–21.9	0.44 (0.18–1.12)	0.134
** **Third	90	21 (23.3)	15.6–33.4	0.93 (0.46–1.89)	0.814

^a^Total number of children evaluated for at least one of the following: microcephaly, CNS imaging abnormalities, Neurologic Abnormalities, Ophthalmologic abnormalities.

^b^The denominator varies according to the missing values for each outcome.

A total of 14 children out of 277 had neurological alterations; 10 were born to women positive to ZIKV infection, 2 born to a woman who had unspecified flavivirus infection, one born to a woman with inconclusive results and one whose mother did not have laboratory evidence of ZIKV infection (Table 3). The later presented convulsions. Among those 10 born to women positive to ZIKV infection, 7 children had only neurological alterations; two children had neurologic alterations and microcephaly (one with alteration of the optic nerve and orthopaedic alterations). Alteration of the optic nerve was also observed in one child with no microcephaly.

The absolute risk of neurologic alterations among children born to women positive to ZIKV infection was 4.6% (CI-95%: 2.5–8.4%) (Table 3). Taking the alterations separately the risk of altered level of consciousness/behaviour, localized motor deficit, convulsion, inadequate visual response and sign of pyramidal release was less than 2%, while the risk of tonus alteration was slightly higher (3.5%, CI-95%: 1.6–7.6%).

A total of 15 out 214 children born to non-negative mothers had ophthalmologic alterations compatible with ZIKV infection, corresponding to an absolute risk of 7% (CI-95%: 4.2% to 11.3%) (Table 3). Taking each of the alterations separately, the risk ranged from 1% (strabismus, nystagmus) to 4.2% (optic nerve).

The absolute risk of dysphagia among children born to women positive to ZIKV infection was around 2% (Table 3). One of these children had microcephaly, neurologic, ophthalmologic alterations and imaging abnormalities (identified in the TF-US, computed tomography and magnetic resonance). Another had neurologic and ophthalmologic alterations. In the two others no other alterations were detected.

A total of 253 children undergone a hearing screening evaluation and 11 failed, none of the later were born to ZIKV negative mothers. However, nine of these children were retested and they all passed in the second evaluation.

There was no statistically significant difference in the risk of adverse outcomes between children born to mothers positive or with suspected Zika exposure and children born to negative mothers.

The percentage of children that had at least one of the abnormalities compatible with CZS was around 20% (Table 5), considering either children born to mothers who were positive, or non-negative (adding mothers with unspecified flavivirus or inconclusive results). The intersection between these different abnormalities did not exceed 1%.

**Table 5:** Absolute risk of Zika related adverse outcomes in the MERG Pregnancy Cohort in Pernambuco, Brazil (2015–2017).

Adverse outcomes	Number of children affected/Total	Absolute risk (%)	95%-CI Absolute risk
**Any of the outcomes**			
**Microcephaly and/or CNS imaging abnormalities and/or Neurologic Abnormalities and/or Ophthalmologic Abnormalities**			
**At first evaluation**			
** **Positive	21/99	21.2	14.1–30.5
** **Positive + Flavivirus + inconclusive	26/119	21.8	15.2–30.3
**At any time**			
** **Positive	28/99	28.3	20.2–38.1
** **Positive + Flavivirus + inconclusive	37/119	31.1	23.3–40.1
**Combined outcomes**			
**Microcephaly and CNS imaging abnormalities and Neurologic Abnormalities**			
**At first evaluation**			
** **Positive^a^	1/121	0.8	0.1–5.8
** **Positive + Flavivirus + inconclusive	1/141	0.7	0.1–5.0
**At any time**			
** **Positive^a^	2/121	1.6	0.4–6.5
** **Positive + Flavivirus + inconclusive	2/141	1.4	0.3–5.6
**Microcephaly and Neurologic Abnormalities and Ophthalmologic Abnormalities**			
**At first evaluation**			
** **Positive	0/180	-	-
** **Positive + Flavivirus^b^ + inconclusive	1/207	0.5	0.1–3.4
**At any time**			
** **Positive	0/180	-	-
** **Positive + Flavivirus^b^ + inconclusive	1/207	0.5	0.1–3.4
**CNS imaging abnormalities and Neurologic Abnormalities**			
**At first evaluation**			
** **Positive^c^	1/120	0.8	0.1–5.8
** **Positive + Flavivirus + inconclusive	1/140	0.7	0.1–5.0
**At any time**			
** **Positive^c^	1/120	0.8	0.1–5.8
** **Positive + Flavivirus + inconclusive	1/140	0.7	0.1–5.0
**Neurologic Abnormalities and Ophthalmologic Abnormalities**			
**At first evaluation**			
** **Positive	1/180	0.5	0.1–3.9
** **Positive + Flavivirus + inconclusive	3/207	1.4	0.5–4.4
**At any time**			
** **Positive	3/180	1.7	0.5–5.1
** **Positive + Flavivirus + inconclusive	5/207	2.4	1.0–5,7

*Total number of children evaluated for microcephaly, CNS imaging abnormalities, Neurologic Abnormalities, Ophthalmologic Abnormalities.

^**a**^Limited.

^**b**^Flavivirus.

^**c**^Robust

Approximately two-thirds of the pregnant women notified with rash belonged to the lower social classes and only a small proportion had higher education (Table 1). There was no association between Zika related adverse outcomes and socioeconomic conditions (S2 Table).

Infection by other arbovirus or TORCH during pregnancy by ZIKV diagnostic status are presented in S3 Table. The frequency of TORCH was below 1%. Approximately 10% tested IgM positive for Dengue and 30% for Chikungunya.

## Discussion

The absolute risk of occurrence of microcephaly in children born to non-negative mothers (positive and suspected) was approximately 3%. Most of the children with microcephaly who had a TF-US performed had no brain abnormality detected. The risk of calcifications and/or ventriculomegaly was 5.7%, of neurologic alterations was 4.6%, of ophthalmologic abnormalities was 6.3% and of dysphagia was around 2%. The percentage of children that had at least one of the abnormalities compatible with CZS was around 20%. However, the intersection between these different abnormalities (microcephaly, imaging, neurologic and ophthalmologic) did not exceed 1%.

The frequency of microcephaly is consistent with the results of other studies [6–11]. The frequency of at least one type of alteration compatible with prenatal exposure to ZIKV infection in our study lies between the 6% and 46% found by Hoen et al. [7] and Brasil et al. [6], respectively. However the comparability between studies is limited by the different clinical and laboratory criteria adopted to define exposure [6,11,27], the limitation of the diagnostic tests to detect ZIKV infection [18] and by the lack of standardisation to define and assess the outcomes [6–13]. Investigation of children in the different studies was similar in terms of microcephaly and imaging, but differed with other outcomes.

Our results suggest that there is not a single presentation of microcephaly, occurring with or without imaging abnormalities and with the presence, or not, of neurological, and ophthalmologic alterations. In Brasil’s cohort the four children with microcephaly had imaging abnormalities, half of them had disproportionate microcephaly [6]. However microcephaly without imaging abnormalities was also described in the French Territories [7], in the U.S. territories and freely associated states [10] and in the continental US and Hawaii [11]. The finding of microcephaly in a child born to a mother with no laboratory evidence of ZIKV infection may be due to false negative result, considering the limitations of the diagnostic tests available [18], and the fact that it occurred during the microcephaly epidemic by Zika [1]. However, another congenital infection cannot be excluded as this mother had rash during pregnancy and was not tested for TORCH. Microcephaly has several causes and is a rare event [28]. The annual mean of occurrence of cases in the state of Pernambuco in the period before the epidemic was 9 [1,29]. We did not investigate genetic and metabolic disorders and they cannot be discarded but are not likely to have occurred.

In our study the imaging abnormalities potentially associated with ZIKV infection were mainly calcifications and ventriculomegaly. A wider variation of abnormalities, particularly in children with microcephaly, has been registered [30] but calcifications and ventriculomegaly were among the most frequent. Intracranial calcifications may occur in other congenital infections like herpes simplex virus, cytomegalovirus and toxoplasmosis and excluding other aetiologies based on specific mother testing is not without problem.

The risk of neurologic alterations among children born to women with laboratory evidence of ZIKV infection during pregnancy was of 5% (CI-95%: 2.7–9.1%) occurring either isolated or in the presence of other type of alterations. The frequency was much lower than that of 26% described by Brasil et al. [6]. One possible explanation for this difference would be that in the study of Brasil et al. [6] children were evaluated at birth and, in our study, they were evaluated later; it is possible that some of these neurologic abnormalities described in the former study were transient.

We found a risk of ophthalmologic alterations of 6.3% (CI-95%: 3.1%-9.5%), very similar to the 6% of the cohort of Rio de Janeiro [6], the only other cohort which provides this information. The ocular abnormalities we found had been described previously, notably in children with microcephaly [31] but also in children without microcephaly [32].

The risk of dysphagia among children born to women with laboratory evidence of ZIKV infection during pregnancy was around 2%, found in children with or without other alterations. Hearing loss and dysphagia associated with congenital virus infection has been previously described, but only in children with microcephaly [33,34].

When we compared the frequency of abnormalities at birth or first evaluation with those that were observed at any time during the follow-up, we found that there was an increase only for the neurological abnormalities. It is not surprising as ophthalmologic abnormalities and dysphagia were already present at the age when the children were evaluated for the first time (median age of 4.65 months (IQR: 2.9–13.4)). However, some neurological abnormalities can be more easily identified or may appear as children get older; for example, epilepsy, motor abnormalities and maturational milestones may not be detectable until later ages.

Overall, we found no statistically significant differences in the risks of adverse outcomes in children born to mothers with non-negative or negative ZIKV test results. However, these results need to be interpreted with caution as the latter group may not be truly negative for ZIKV infections. As this is a cohort of PW notified with rash, all participants had symptoms compatible with ZIKV infection during an active epidemic. In fact, in addition to rash, in the negative group, almost 80% of the mothers reported fever, arthralgia or fatigue and more than 60% reported myalgia, among other symptoms. Limitations of the accuracy of the diagnostic tests for ZIKV and the narrow time window in which the molecular tests remain positive [18] also increase the uncertainty in excluding this diagnosis [18]. Another point to be considered is that, due to financial and operational constrains, we prioritized the evaluation of children born to mothers who had laboratory evidence of ZIKV infection during pregnancy. Therefore, the number of children born to mothers who tested negative is small, especially in relation to neurologic and ophthalmologic assessments, and therefore the power to detect differences in the risk of abnormalities between children born to mothers of this group and those born to non-negative mothers is low. In summary, false negative results and lack of power may underly the lack of association between adverse outcomes and positive ZIKV test results.

The definition of CZS is still being built and the clinical features of the less severe cases are not well known. Our results suggest that abnormalities occur more frequently isolated than in combination. The frequency of any adverse outcome could be as high as one in five children whose mother was infected during pregnancy but the frequency of the concomitant presentation of microcephaly, imaging, neurologic and ophthalmologic abnormalities was very low. Even for the children with microcephaly, there was a degree of variation, including children with no other abnormality detected. The reason for differences in the presentation needs to be further explored. It would be expected more severe cases in children born to mothers infected in the first trimester of pregnancy as observed in some studies [7,8,11] and in other congenital diseases [35,36]. However, abnormalities were also found in children born to mothers who were infected later in pregnancy. Another point to learn the full spectrum is that some neurodevelopmental abnormalities, which may be an important component of this Syndrome, will only be better evaluated later in these children.

As pregnant women were notified because of presence of rash this symptom was reported in the interview by 98% of them. The characteristics of the rash did not differ between those who had or not laboratory evidence of ZIKV infection and, in general, it was not possible to distinguish Zika infection just by clinical characteristics. The proportion of laboratory evidence of DENV and CHIKV infection among those who had no laboratory evidence of Zika was small.

Five infant deaths occurred after delivery, three of which, including two children with Down Syndrome, in children with severe cardiac malformation which are not likely to be related with ZIKV infection. For the other foetal or infant deaths it is not possible to evaluate whether ZIKV infection was a contributing factor.

Overall, the cohort had a low socioeconomic status, with approximately two-thirds of participants belonging to the lower social classes and only a small proportion of the mothers having higher education. An ecological study carried out by MERG showed that there was a strong association between a higher prevalence of microcephaly and poor living conditions in the Metropolitan Region of Recife [37]. In fact, this area is characterized by high social inequality, and the pattern of distribution of several infectious diseases reflects the uneven social conditions [38,39].

The frequency of TORCH (Toxoplasmosis, Rubella, Cytomegalovirus and Parvovirus) infections among the pregnant women was low, but at least one-third of those tested for Chikungunya were positive. We note that Zika and Chikungunya viruses were co-circulating in this area in the same period and the neurological manifestations of the dual infection in the adult population has been recently described [40]. We cannot rule out that CHIKV may be playing a role in some of the outcomes we found.

This study has some strengths and limitations. The partnership with health authorities by using the notification system in the recruitment of pregnant women with rash in the context of the public health emergency reduced the risk of selection bias. There was a higher health-seeking behaviour increasing the sensitivity in detecting women with rash. The use of different laboratory technics and sequenced tests allowed to compare hierarchical groups of evidence of ZIKV infection in a sensitive analysis. The use of longitudinal molecular and serologic testing enhanced diagnostic sensitivity for defining cases of ZIKV infection among pregnant women presenting with rash and allowed us to detect a larger number of infected women. Another strength was the standardized and deep investigation of infant outcomes, performed by experienced specialists.

The children were not evaluated at birth and the time elapsed until the first evaluation varied. Early transient symptoms may not have been detected, but the adverse outcomes which were diagnosed are likely to be persistent. Furthermore, as impairments may manifest at a later age, some abnormalities were only detected because of the delayed evaluation. Later manifestations point to the need to follow these children for a longer period.

Asymptomatic pregnant women were not included and it is possible that risk estimates for children born to asymptomatic pregnant women could differ but there is no evidence to date that it occurs [8,41].

Misclassification in relation to exposure may have happened due to the limitations of the laboratory tests, the short duration of viremia and the overlapping of the clinical features with other arbovirus infections and TORCHs. However, there was no marked difference in the frequency of adverse outcomes between the groups with different levels of evidence. Errors of classification may also have occurred in the unexposed group because they were symptomatic and therefore could have another infection.

Not all children were evaluated for all outcomes. However, as the children were not selected to the comprehensive evaluation for any particular reason, it may have decreased the sample size but it is not likely to have introduced any selection bias.

We may not exclude that there may have been some abnormalities noted in the infants born to mothers with laboratory evidence which have an aetiology unrelated to ZIKV. Other studies with an adequate control group of unexposed women would allow the estimation of the attributable risk.

## Conclusions and recommendations

The frequency of microcephaly in our results is consistent with previous studies. In addition, as the children were evaluated at a later age and repeatedly by different groups of specialists our findings provide new insight into the absolute risk of more specific abnormalities, especially those more likely to be persistent, and suggest that isolated manifestations occur more frequently than in combination. Due to the rare nature of some outcomes and the possibility of later manifestations of some components of the syndrome, large scale individual participant data meta-analysis to explore subgroups and the long term evaluation of children are imperative to identify the full spectrum and to plan actions to reduce the damages of the Congenital Zika Syndrome. For the children with microcephaly, we recommend a comprehensive clinical examination by the paediatrician to identify other adverse outcomes/complications and the early initiation of neuropsychomotor stimulation. For the other exposed but symptomatic or asymptomatic children, we recommend screening for neurologic and ophthalmologic abnormalities and developmental delay.

## Supporting information

S1 Table Characteristics of women with rash by ZIKV diagnostic status in the MERG Pregnancy Cohort in Pernambuco, Brazil (2015–2017). (DOCX)

S2 Table Socioeconomic conditions and risk of Zika related adverse outcomes in the in the Zika positive group of the MERG Pregnancy Cohort in Pernambuco, Brazil (2015–2017). (DOCX)

S3 Table Infection by other arbovirus or TORCH during pregnancy by ZIKV diagnostic status in the MERG Pregnancy Cohort in Pernambuco, Brazil (2015–2017). (DOCX)
