# Supplementary material for: Zika-related adverse outcomes in a cohort of pregnant women with rash in Pernambuco, Brazil
Source: PLoS Negl Trop Dis. 2021 Mar 8;15(3):e0009216. doi: 10.1371/journal.pntd.0009216 (PMC7971861; doi:10.1371/journal.pntd.0009216)
Supplement: S1 Table — (DOCX) [file pntd.0009216.s001.docx]

**S1 Table. Characteristics of women with rash by ZIKV diagnostic status in the MERG Pregnancy Cohort in Pernambuco, Brazil (2015-2017).**

|  | **ZIKV-positive** | **Suspected ZIKV exposure** | | **ZIKV-negative**  **(n = 127)** | ***P value^a^***  *Positive v. negative* | ***P value^b^*** *Positive+Suspected v. negative* |
| --- | --- | --- | --- | --- | --- | --- |
|  | **Robust + Moderate + Limited (n = 277)** | **Unspecified flavivirus-positive (n = 78)** | **Inconclusive**  **(n = 19)** |  |  |  |
|  | **N (%)** | | | |  |  |
| **Sociodemographic factors** |  |  |  |  |  |  |
| **Age, years** | 26.4 (7.7) | 24.5 (5.7) | 27.8 (7.8) | 26.4 (6.3) | 0.96 | 0.69 |
| **Race/ethnicity**  “Preto” (ie, black)  “Pardo” (ie, mixed race)  “Branco” (ie, white)  Other  *Missing* | 24 (8.7)  191 (69.0)  60 (21.6)  2 (0.7)  *0* | 6 (7.7)  56 (71.8)  16 (20.5)  0 (0)  *0* | 0 (0)  15 (79.0)  3 (15.8)  1 (5.3)  *0* | 20 (15.9)  73 (57.9)  28 (22.2)  5 (4.0)  *1* | 0.01 | 0.003 |
| **Years of education**  0-7  8-10  11+ | 109 (39.4)  140 (50.5)  28 (10.1) | 29 (37.2)  38 (48.7)  11 (14.1) | 7 (36.9)  10 (52.6)  2 (10.5) | 36 (28.3)  75 (59.1)  16 (12.6) | 0.10 | 0.11 |
| **Social Class**  A, B1, and B2  C1  C2  D and E | 25 (9.0)  40 (14.4)  109 (39.3)  103 (37.2) | 9 (11.5)  14 (18.0)  25 (32.0)  30 (38.5) | 1 (5.3)  2 (10.5)  9 (47.4)  7 (36.8) | 12 (9.4)  34 (26.8)  43 (33.9)  38 (29.9) | *0.03* | *0.03* |
| **Monthly per capita family income, BRL (Median (IQR))** | 1072 (800 – 1800) | 1200 (850 – 1880) | 1200 (880 – 1860) | 1600 (880 – 2340) | *0.004* | *0.007* |
| **Reproductive history** |  |  |  |  |  |  |
| **Previous pregnancy**  Yes  No | 189 (67.9)  89 (32.1) | 43 (55.1)  35 (44.9) | 14 (73.7)  5 (25.0) | 75 (59.1)  52 (40.9) | *0.08* | *0.17* |
| **Children with malformations from previous pregnancies**  Yes  No  N/A^a^ | 4 (1.5)  163 (58.8)  110 (39.7) | 3 (3.8)  35 (44.9)  40 (51.3) | 1 (5.3)  13 (68.4)  5 (26.3) | 4 (3.1)  67 (52.8)  56 (44.1) | *0.32* | *0.67* |
| **Current pregnancy characteristics** |  |  |  |  |  |  |
| **Smoking**  Yes  No | 17 (6.1)  260 (93.9) | 11 (14.1)  67 (85.9) | 1 (5.3)  18 (94.7) | 9 (7.1)  118 (92.9) | *0.72* | *0.81* |
| **Illicit drug use**  Yes  No | 3 (1.1)  274 (98.9) | 2 (2.6)  76 (97.4) | 0 (0)  19 (100) | 2 (1.6)  125 (98.4) | *0.81* | *0.92* |
| **Delivery mode**  Vaginal  Cesarean  Forceps  *Missing* | 141 (51.6)  131 (48.0)  1 (0.4)  *3* | 38 (48.7)  40 (51.3)  0 (0)  *0* | 12 (63.2)  7 (36.8)  0 (0)  *0* | 75 (60.5)  49 (39.5)  0 (0)  *4* | *0·22* | *0·17* |

^a^Comparison of the ZIKV-positive group versus the ZIKV-negative group: *P* values for categorical variables are from Chi-squared tests or, for variables with cells including ≤5 observations, Fisher’s exact tests; *P* values for continuous variables are from Mann-Whitney U tests. ^b^Comparison of the positive and suspected exposure group (i.e., ZIKV-positive, unspecified flavivirus positive, and inconclusive groups) versus the ZIKV-negative group: *P* values for categorical variables are from Chi-squared tests or, for variables with cells including ≤5 observations, Fisher’s exact tests; *P* values for continuous variables are from Mann-Whitney U tests.
