# Supplementary material for: Zika-related adverse outcomes in a cohort of pregnant women with rash in Pernambuco, Brazil
Source: PLoS Negl Trop Dis. 2021 Mar 8;15(3):e0009216. doi: 10.1371/journal.pntd.0009216 (PMC7971861; doi:10.1371/journal.pntd.0009216)
Supplement: S2 Table — (DOCX) [file pntd.0009216.s002.docx]

**S2 Table. Socioeconomic conditions and risk of Zika related adverse outcomes in the in the Zika positive group of the MERG Pregnancy Cohort in Pernambuco, Brazil (2015-2017).**

| **Socioeconomic variables** | **Number of**  **children** | **Number of cases (%)** | **95%-CI** | **Relative Risk**  **(95%-CI)** | **p-value** |
| --- | --- | --- | --- | --- | --- |
| **Microcephaly** | 278 | 7 | - | - | - |
| **Years of education** |  |  |  |  |  |
| 0 - 8 | 70 | 3 (4.3) | 1.3 –12.8 | Reference | - |
| 9 - 11 | 83 | 1 (1.2) | 0.2 – 8.4 | 0.28 (0.03 – 2.6) | 0.332 |
| 12+ | 125 | 3 (2.4) | 0.8 – 7.3 | 0.50 (0.05 – 4.7) | 1.000 |
| **Social class** |  |  |  |  |  |
| A, B1 e B2 | 25 | 0 (0.0) | - | - | - |
| C1 | 40 | 4 (10.0) | 3.6 – 24.6 | 5.1 (0.98 – 27.0) | 0.052 |
| C2 | 110 | 1 (0.9) | 0.1 – 6.3 | 0.47 (0.04 – 5.1) | 0.611 |
| D - E | 103 | 2 (1.9) | 0.5 – 7.6 | Reference | - |
| **Any of the abnormalities*** | 185 | 36 | - | - | - |
| **Years of education** |  |  |  |  |  |
| 0 - 8 | 51 | 10 (19.6) | 10.6 – 33.3 | Reference | - |
| 8 - 11 | 54 | 13 (24.1) | 14.3 – 37.7 | 1.23 (0.59 – 2.5) | 0.641 |
| 12+ | 80 | 13 (16.2) | 9.6 – 26.3 | 0.83 (0.39 – 1.74) | 0.643 |
| **Social class** |  |  |  |  |  |
| A, B1 e B2 | 19 | 2 (10.5) | 2.3 – 97.7 | Reference | - |
| C1 | 25 | 5 (20.0) | 8.0 – 41.7 | 1.90 (0.41 – 8.75) | 0.680 |
| C2 | 71 | 14 (19.7) | 11.9 – 30.9 | 1.87 (0.46 – 7.54) | 0.507 |
| D - E | 70 | 15 (21.4) | 13.2 – 32.9 | 2.04 (0.51 – 8.13) | 0.347 |

*Total number of children evaluated for at least one of the following: microcephaly, CNS imaging abnormalities, Neurologic Abnormalities, Ophthalmologic Abnormalities
