# Supplementary material for: Zika-related adverse outcomes in a cohort of pregnant women with rash in Pernambuco, Brazil
Source: PLoS Negl Trop Dis. 2021 Mar 8;15(3):e0009216. doi: 10.1371/journal.pntd.0009216 (PMC7971861; doi:10.1371/journal.pntd.0009216)
Supplement: S3 Table — (DOCX) [file pntd.0009216.s003.docx]

**S3 Table. Infection by other arbovirus or TORCH during pregnancy by ZIKV diagnostic status in the MERG Pregnancy Cohort in Pernambuco, Brazil (2015-2017).**

| **Infection** | **Total** | **ZikV-Positive** | **Suspected ZIKV exposure** | | **Positive + Flavivírus + inconclusive** | **ZikV-Negative** | **p-valor*** | **p-valor**** |
| --- | --- | --- | --- | --- | --- | --- | --- | --- |
|  |  |  | **Flavivirus** | **Inconclusive** |  |  |  |  |
| TORCH | N/tested(%) |  |  |  |  |  |  |  |
| Rubella |  |  |  |  |  |  |  |  |
| IgM | 0/303 (0) | 0/185 (0) | 0/36 (0) | 0/6 (0) | 0/227 (0) | 0/76 (0) | - | - |
| IgG | 259/295 (87·8) | 163/179 (91.1) | 28/35 (80.0) | 4/6 (70.0) | 195/220 (88.6) | 64/75 (85.3) | 0.177 | 0.450 |
| Cytomegalovirus |  |  |  |  |  |  |  |  |
| IgM | 1/307 (0.3) | 1/187 (0.5) | 0/36 (0) | 0/6 (0) | 1/229 (0.4) | 0/78 (0) | 1.000^a^ | 1.000^a^ |
| IgG | 289/305 (94.7) | 175/185 (94.6) | 34/36 (94.4) | 6/6 (100) | 215/227 (94.7) | 74/78 (94.9) | 0.927 | 0.957 |
| DNA | 4/68 (5.9) | 4/39 (10.3) | 0/16 (0) | 0/1 (0) | 4/56 (7.1) | 0/12 (0) | 0.561^a^ | 1.000^a^ |
| Parvovirus |  |  |  |  |  |  |  |  |
| IgM | 2/296 (0.7) | 1/183 (0.5) | 0/34 (0) | 0/6 (0) | 1/223 (0.4) | 1/73 (1.3) | 0.490^a^ | 0.433^a^ |
| IgG | 216/292 (74.0) | 133/178 (74.7) | 24/33 (72.7) | 3/6 (50) | 160/217 (73.7) | 56/75 (74.7) | 0,993 | 0,874 |
| Toxoplasmosis |  |  |  |  |  |  |  |  |
| IgM | 1/277 (0.4) | 1/167 (0.6) | 0/33 (0) | 0/5 (0) | 1/205 (0.5) | 0/72 (0) | 1.000^a^ | 1.000^a^ |
| IgG | 188/277 (67.9) | 112/167 (67.1) | 24/33 (72.7) | 5/5 (100) | 141/205 (68.8) | 47/72 (65.3) | 0.788 | 0,584 |
| **Arboviruses** |  |  |  |  |  |  |  |  |
| Chikungunya |  |  |  |  |  |  |  |  |
| IgM | 94/320 (29.4) | 61/196 (31.1) | 11/40 (27.5) | 1/6 (16.7) | 73/242 (30.2) | 21/78 (26.9) | 0.493 | 0.585 |
| PCR | 39/54 (72.2) | 25/34 (73.5) | 6/6 (100) | 1/1 (100) | 32/41 (78.0) | 7/13 (53.8) | 0.295^a^ | 0.152^a^ |
| Dengue |  |  |  |  |  |  |  |  |
| IgM | 37/317 (11.7) | 26/196 (13.3) | 2/38 (5.3) | 1/6 (16.7) | 29/240 (12.1) | 8/77 (10.4) | 0.517 | 0.687 |
| IgG | 104/108 (96.3) | 104/108 (96.3) | - | - | 104/108 (96.3) | - | - | - |

* Zika negative versus ZikV.

** Zika negative versus Positive + Flavivírus + inconclusive

^a^ Fisher exact test.
